# A comprehensive dataset of 32 million pentapeptide structures for high-throughput virtual screening

**DOI:** 10.1038/s41597-026-07838-4

**Published:** 2026-07-07

**Authors:** Josep-Ramon Codina, Emre Dikici, Sapna K. Deo, Sylvia Daunert

**Affiliations:** 1 Department of Biochemistry and Molecular Biology, University of Miami, Miller School of Medicine, 1600 NW 10th Ave, Miami, FL 33136 USA; 2 Dr. John T. Macdonald Foundation Biomedical Nanotechnology Institute, 1951 NW 7th Ave#475, Miami, FL 33136 USA; 3 Sylvester Comprehensive Cancer Center, University of Miami, 1475 NW 12th Ave, Miami, FL 33136 USA

## Abstract

Small peptides are widely used as binders, modulators, and structural motifs, but their conformational flexibility complicates structure-based analysis and high-throughput screening. We present an open dataset of three-dimensional structures for the complete space of canonical amino-acid pentapeptides: 3,200,000 unique sequences with up to 10 conformers per sequence, for a total of 32,000,000 peptide conformers. Structures were generated directly from sequence using an automated workflow built on UCSF ChimeraX for model construction, Reduce for hydrogen placement, and RDKit for conformer generation and optimization. The dataset is distributed as compressed archives with an accompanying index that maps each sequence and conformer identifier to its coordinate record, enabling efficient download, subset selection, and programmatic access. Technical validation includes symmetry-aware inter-conformer RMSD analysis, Ramachandran quality assessment, and benchmarking against experimentally observed pentapeptide fragments from the Protein Data Bank. Although we focus here on pentapeptides to enable exhaustive sequence coverage, the publicly released workflow is solely based on open-source software and can be applied to other short peptides to generate comparable conformer libraries. This resource supports virtual screening with pre-generated peptide conformer ensembles, method benchmarking, and machine-learning applications in peptide design and protein engineering by removing the need for researchers to repeatedly generate large conformer ensembles from scratch.

## Background & Summary

Peptides are fundamental components in many biological functions acting as binders and modulators, for example, as hormones, neurotransmitters, antibiotics, or signaling molecules. Their versatility and specificity make them attractive candidates for therapeutic applications^1,2^. The number of FDA-approved peptide therapeutics has experienced a 4-fold increase since 2018, reflecting growing interest in their clinical potential^3–10^. However, they still represent only ~8% of all approved drugs^10^. Understanding the structural and dynamic properties of peptides is critical for elucidating their biological roles and for the rational design of peptide-based therapeutics^11,12^. Accurately modeling peptides poses significant challenges due to their intrinsic flexibility and the vast conformational space that they can occupy^13^. Traditional experimental methods like X-ray crystallography, cryogenic electron microscopy (Cryo-EM), and NMR spectroscopy provide detailed structural information, but are often time-consuming and not suitable for high-throughput analysis^14–16^. Computational modeling offers a viable alternative, enabling the exploration of peptide structures and dynamics efficiently.

Recent breakthroughs in protein structure prediction, particularly the development of AlphaFold, have revolutionized the field by achieving unprecedented accuracy in modeling protein structures from amino acid sequences^17^. However, many widely used predictors are optimized for accuracy rather than throughput, and they are not practical for enumerating and sampling conformational ensembles across extremely large peptide libraries^17,18^. In addition, accessibility can be limited by hardware requirements, platform constraints, and usage caps in hosted offerings. For large-scale applications, the bottleneck is not the ability to predict one peptide, but the ability to generate many peptide ensembles reliably, reproducibly, and at low computational cost. While cloud-based alternatives exist, they impose usage restrictions, that make it difficult to obtain large datasets^17,19^.

Several established peptide modeling tools address de novo folding and low-energy structure prediction, including PEP-FOLD, Rosetta, and PEPstrMOD. These methods can perform well for individual peptides, but they are typically less suitable for industrial-scale generation across millions of sequences, particularly when the goal is to produce standardized conformer ensembles for downstream screening^20–22^. In contrast, conformer-generation approaches developed for small molecules, such as RDKit (and commercial tools including OMEGA and ConfGen), provide efficient sampling of multiple geometries using distance geometry combined with force-field minimization^23–25^. RDKit, in particular, combines Experimental-Torsion Knowledge Distance Geometry (ETKDG) with force-field-based minimization to generate realistic conformers with favorable geometries. Recent benchmarking studies have validated RDKit’s ability to capture low-energy structures with high efficiency, often outperforming more complex folding algorithms in terms of speed and ensemble diversity^26^.

**Fig. 1:**
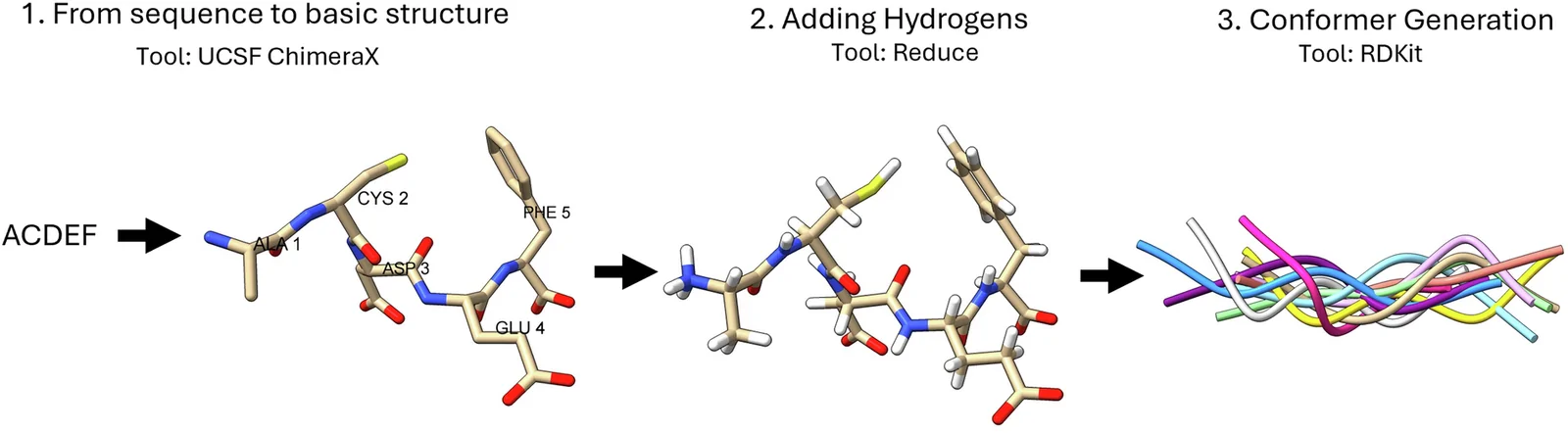
Overview of the Automated Peptide Structure Generation Workflow. This figure shows the three-step process for systematically generating small peptide structures. First, UCSF ChimeraX constructs a basic model from the sequence (example: pentapeptide ACDEF). Next, Reduce carries out the hydrogen placement. Finally, RDKit generates multiple low-energy conformers, capturing peptide flexibility.

Herein we present an open dataset of peptide conformational ensembles together with the reproducible pipeline used to generate them. The pipeline integrates UCSF ChimeraX, Reduce, and RDKit to construct peptide models from sequence, optimize hydrogen placement, generate and minimize conformers at scale (Fig. 1). Using this workflow, we generated an extensive database comprising all possible pentapeptide sequences (20^5^ = 3.2 million) with ten conformers each, resulting in 32 million peptide structures. Both the dataset and the workflow are released publicly, enabling reuse of the current dataset and straightforward extension to other short peptides^23,27,28^.

To demonstrate the scalability of the approach and to provide a broadly reusable resource, we focus on pentapeptides. Pentapeptides provide a practical balance between sequence complexity and manageability. Their modest length allows for complete enumeration of all possible sequences using natural amino acids, while still capturing a rich diversity of backbone and sidechain conformations. This makes them particularly suitable for high-throughput applications where scalability and conformational flexibility are essential^29,30^. At the same time, longer peptides, cyclic peptides, and peptides containing noncanonical residues are highly relevant in contemporary peptide discovery, where cyclization and chemical expansion are often used to improve conformational control, stability, permeability, and functional diversity^31–34^. In principle, the workflow described here could be extended to such systems; however, these extensions are not trivial, because noncanonical residues require additional parameterization and residue definitions, while cyclic topologies and expanded chemical alphabets substantially increase both structural complexity and the size of the accessible sequence space^33–35^. We therefore regard the present canonical linear pentapeptide library as a deliberately tractable and fully enumerable foundation for large-scale peptide modeling. For this initial release, we distribute structures in PDB format because of its broad compatibility with current docking and visualization tools for short canonical peptides. We note, however, that PDBx/mmCIF is the more extensible wwPDB standard and may be advantageous for future libraries incorporating noncanonical residues or more complex chemistries^35,36^.

By providing access to these pre-generated peptide models, researchers can bypass the need for computationally intensive structure generation, thereby accelerating the pace of discovery and enabling more efficient investigation into peptide functions and interactions. This makes our dataset and method particularly valuable for machine learning or high-throughput studies where processing large libraries quickly and cost-effectively is essential. The primary audience for this resource is researchers performing high-throughput peptide modeling, virtual screening, method benchmarking, and machine-learning-guided peptide discovery, particularly in settings where standardized conformer ensembles across large peptide collections are needed^37,38^.

## Methods

### Automated peptide construction

The library was generated by enumerating all possible pentapeptide sequences using the 20 natural amino acids, resulting in a total of 3,200,000 unique sequences. We developed a Python script interfacing with UCSF ChimeraX (v1.8) to automate the generation of initial structures. In this initial step, UCSF ChimeraX was used to generate a single structure per peptide, assigning an extended conformation dihedral angle (φ = −60.0°, ψ = 120.0°) as the starting conformer. This guaranteed consistency across all generated peptide models while minimizing manual effort.

**Fig. 2:**
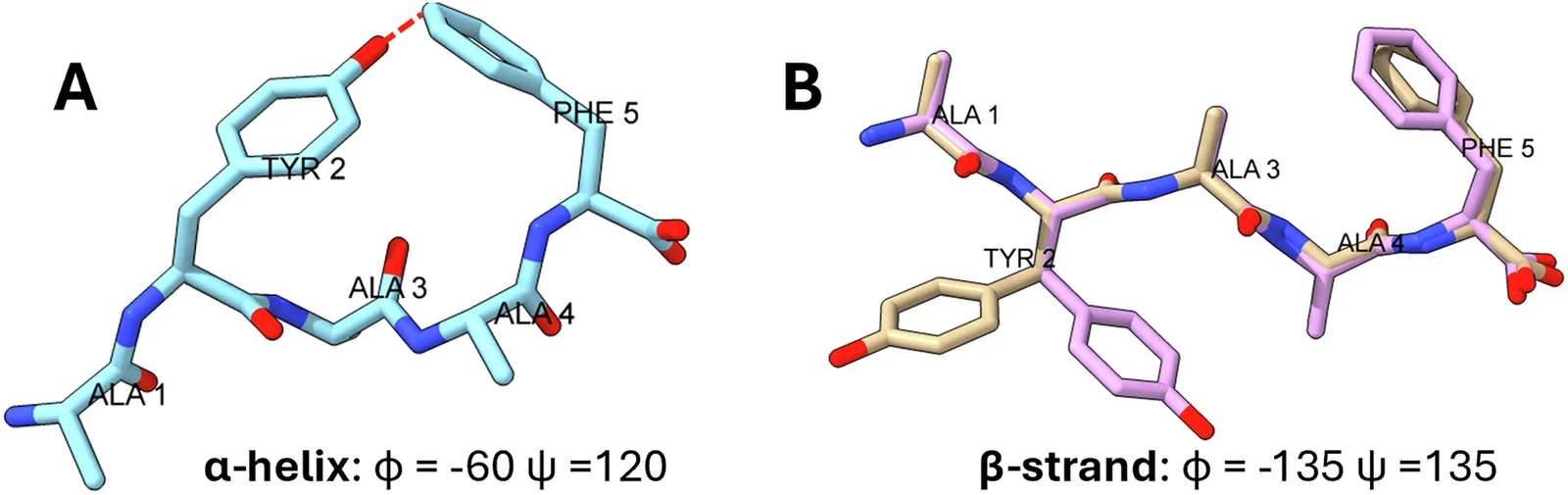
Structural Impact of Backbone Dihedral Angles on Peptide Conformation. (**A**) A pentapeptide built using an extended conformation dihedral angles (ϕ= −60°, ψ = 120°) shown in cyan. The steric clashes between bulky sidechains (TYR2-PHE5) lead to structural strain and deviation from a stable conformation. (**B**) The same peptide built using β-strand dihedral angles (ϕ = −135°, ψ = 135°) shown in camel. This conformation reduces steric hindrance, leading to a more stable structure. Additionally, an AlphaFold-generated prediction (pink) aligns well with the β-strand model (RMSD = 0.18 Å), supporting the preference for this conformation in some sequences.

Peptides which failed to produce the target number of conformers from the initial backbone were identified and reconstructed using a β-strand-like backbone conformation (φ = −135°, ψ = 135°), (Fig. 2A,B). We record whether the extended or β-strand-like backbone template was used for each sequence in the accompanying metadata, enabling users to subset or audit these cases.

### Hydrogen optimization

Following the generation of initial peptide structures, the Reduce tool was employed to add missing hydrogen atoms and optimize hydrogen bonding networks. We used Reduce’s default settings to ensure proper hydrogen placement and protonation states. Standard physiological protonation states were assigned: Asp and Glu were deprotonated; Lys and Arg were protonated; His residues were protonated at Nδ (HID form); and Cysteine residues were retained in their neutral thiol form (see Table 1). All peptides were modeled as uncapped, zwitterionic termini with a protonated N-terminus (NH3^+^) and a deprotonated C-terminus (COO^−^), as reflected in the output PDBs. This step improves the structural realism of the models, which is critical for accurate molecular interactions in downstream applications. Effects of solubility, aggregation propensity, and other environment-specific solution behaviors were not modeled in the current library.

**Table 1:** Protonation states of Reduce.

Residue	Default Protonation State	Notes
**Asp (D)**	Deprotonated (COO−)	Negatively charged at pH ~7.0
**Glu (E)**	Deprotonated (COO−)	Negatively charged at pH ~7.0
**His (H)**	Neutral (Nδ-H)	Histidine assigned in HID form
**Lys (K)**	Protonated (NH₃+)	Positively charged at pH ~7.0
**Arg (R)**	Protonated (guanidinium NH₂+)	Always positively charged
**Cys (C)**	Neutral (SH)	Maintains thiol group unless disulfide bonds are present
**Tyr (Y)**	Neutral (phenol OH)	Remains protonated at physiological pH

### Conformer generation

Conformer generation and minimization were performed in RDKit (version 2024.09.5). For each peptide, the hydrogenated PDB structure was imported with hydrogens retained (Chem.MolFromPDBFile, removeHs = False), and up to 10 conformers were generated with EmbedMultipleConfs using experimental torsion preferences and basic chemical knowledge (useExpTorsionAnglePrefs = True; useBasicKnowledge = True; other parameters left as RDKit defaults). Each conformer was then energy-minimized using the MMFF94 force field (MMFFGetMoleculeProperties with mmffVariant = “MMFF94”; MMFFGetMoleculeForceField; ff.Minimize). RDKit produced 10 conformers for approximately 99% of sequences; in a small subset enriched in bulky residues (Arg, Trp, Tyr), fewer conformers were obtained due to steric constraints and embedding termination under the default maxAttempts setting.

### Quality analysis of the conformers

We performed three analyses to evaluate conformer quality: inter-conformer RMSD, Ramachandran classification, and comparison to PDB structures. To assess conformational diversity, RMSDs were computed between all pairwise combinations of the 10 conformers generated per peptide using RDKit’s symmetry-aware GetBestRMS function. Structures were loaded with hydrogens removed, so RMSDs were evaluated over heavy atoms only. This approach reduces artificial contributions from methyl rotations, and accounts for symmetry-equivalent atom mappings in side chains. The mean RMSD per peptide was used as a measure of ensemble diversity. Backbone dihedral quality was assessed using phenix.ramalyze (default settings). For each conformer, residues were classified as Favored, Allowed, or Outlier based on φ/ψ angles.

### Comparison to experimental structures

To benchmark the structural realism of the peptides generated by our pipeline, we performed a comparative analysis against experimentally determined structures. Specifically, we extracted all linear pentapeptides (unmodified, non-cyclic) from the PDB and matched each fragment to the library peptide with the same sequence. Because the experimental structures span heterogeneous conditions and include both isolated and context-dependent peptide states, we interpret this benchmark as a test of structural coverage rather than as a strict reproduction of every experimental microenvironment. A total of 228 structures were analyzed.

For each sequence match, we evaluated two complementary similarity metrics. First, we computed a symmetry-aware backbone RMSD between the PDB-derived peptide and each generated conformer using RDKit’s GetBestRMS after removing hydrogens from both structures. Second, we compared backbone torsions (phi, psi, and omega) in torsion space using circular angle differences. For both analyses, the best-scoring conformer, defined as the conformer with the lowest metric value, was retained for each experimental peptide.

Experimentally determined benchmark peptides were identified from the archive-wide PDB SEQRES sequence file *pdb_seqres.txt.gz*, downloaded from the RCSB PDB derived-data archive (RCSB file download services: https://files.rcsb.org/pub/pdb/derived_data/pdb_seqres.txt.gz, accessed May 20, 2025). This file contains the deposited polymer sequences in FASTA format at the chain level. We parsed the FASTA headers to extract the chain identifier, molecule type, and sequence length, then retained only entries annotated as mol:protein with length 5. We further filtered these candidates to sequences composed exclusively of the 20 canonical amino acids.

For each retained candidate, the 4-character PDB accession and chain identifier were extracted from the FASTA header and the corresponding coordinate file was downloaded from the PDB archive. Coordinates were processed in R, where the target chain was isolated, water molecules were removed, and alternate locations were resolved by retaining the blank altLoc when present or otherwise the dominant alternate-location label. Candidates containing non-canonical residues in the extracted coordinates were excluded. The resulting chain was written as a standalone PDB file for downstream comparison to the generated peptide library. Finally, we compared the coordinate-derived residue sequence with the corresponding SEQRES annotation and retained only coordinate-complete pentapeptides whose coordinate and SEQRES sequences matched exactly.

## Data Records

The complete dataset is publicly available in Zenodo^39^. It contains all 3,200,000 canonical pentapeptide sequences (20^5^) with up to 10 conformers per sequence. Structures are provided as PDB coordinate files.

For distribution, the dataset is packaged as 20 zstd-compressed tar archives partitioned by the first residue of the peptide sequence (A.tar.zst, C.tar.zst, E.tar.zst … Y.tar.zst). The total size of the 20 compressed files is 22.1 GB, while the uncompressed dataset is about 206 GB. Each peptide sequence is assigned a folder identifier of the form H_ < sequence > (for example, H_AAAAA), where < sequence > is the one-letter amino-acid sequence used in all tabular files. Within each peptide folder, individual conformers are identified by the concatenation of the folder identifier and a zero-based conformer number, H_ < sequence > _conformer_ < conf_number > (for example, H_AAAAA_conformer_0). These identifiers are used consistently across the coordinate archives and the tabular metadata.

The deposit includes an index file mapping each sequence and conformer identifier to the corresponding archive and internal path, enabling efficient retrieval of subsets without regenerating structures. *Sequence_conformer_location.txt* is the primary lookup table that links each (Sequence, Conformer) pair to its coordinate record. For each conformer, the table reports the corresponding folder name and a Location field that specifies the archive file and internal path needed to retrieve the structure from the compressed tar. *Conformer_metrics.txt* provides per-conformer quality annotations and provenance flags. Fields include Ramachandran classifications (Favored, Allowed, Outlier) with residue-type context, indicators for whether a matching experimental structure exists in the Protein Data Bank (PDB), the matched PDB identifier and the RMSD between the deposited conformer and the matched PDB structure, and generation provenance including the initial conformation used for ChimeraX. *RMSD_compressed_all.zip* contains *RMSD_ALL.txt*, a table of pairwise RMSD values within each peptide. Each row reports Peptide (sequence), Conformer1, Conformer2, RMSD, and a peptide-level category label.

## Technical Validation

### Conformational diversity (RMSD)

**Fig. 3:**
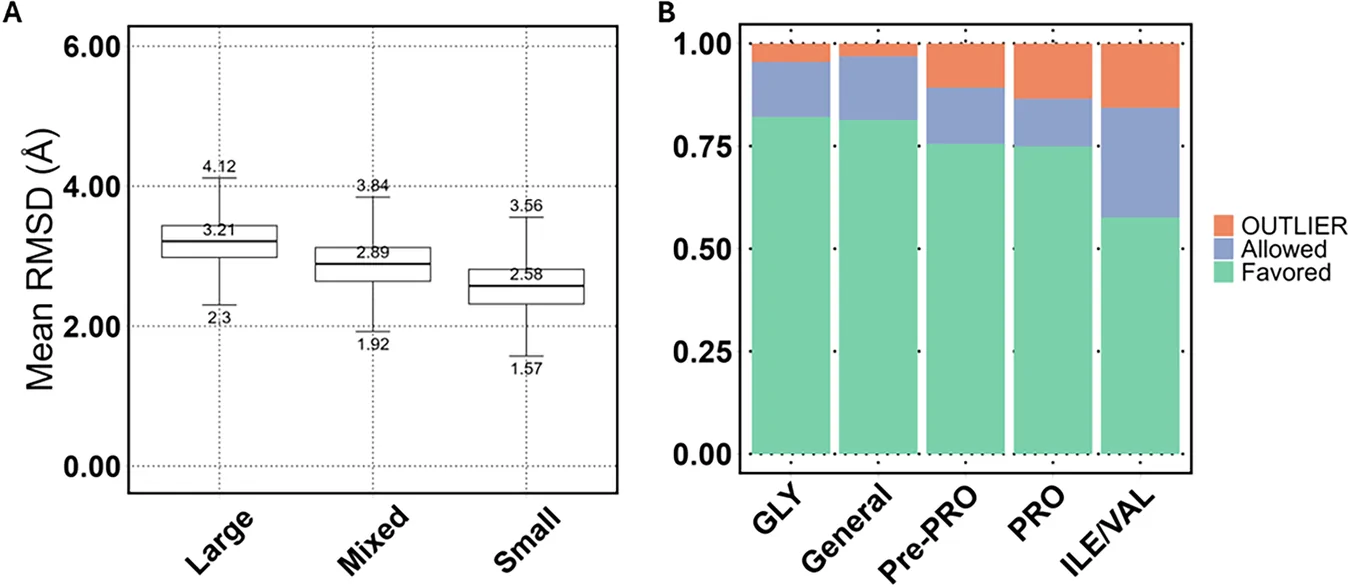
(**A**) Root‐mean‐square distance (RMSD) values (in Å) for three different peptides composed only of small or large residues or a mix of these. (**B**) Ramachandran classification (Favored, Allowed, or Outlier) for various residue types from ramalyze.phenix tool: glycine (GLY), general residues (e.g., Ala, Leu, etc.), proline (PRO), pre-proline (Pre-PRO), and branched aliphatic residues (ILE/VAL). Each bar is normalized to a total height of 1.0, illustrating the fraction of residues that fall into each Ramachandran category for each residue type.

To evaluate structural diversity, we calculated heavy-atom, symmetry-aware root mean squared distance (RMSD) between different conformers of the same sequence using RDKit. Inter-conformer RMSDs were computed for all pairwise combinations of the conformers available for each peptide, and the mean pairwise RMSD per peptide was used as the diversity metric. Peptides composed of small residues (Gly, Ala, Ser) exhibited lower RMSD, while those with bulky, flexible residues (Phe, Trp, Arg) showed higher RMSD, consistent with expected physical properties. This confirms that the generated ensembles capture meaningful conformational diversity (Fig. 3A).

### Backbone stability (Ramachandran Analysis)

Backbone dihedral quality was assessed using the phenix.ramalyze tool with default settings^40^. Most residues (>75%) populated the Favored/Allowed regions of the Ramachandran plot (Fig. 3B). Glycine showed the highest flexibility, while Proline distributions reflected its known constraints. β-branched residues (Ile, Val) showed increased deviation in some conformers due to steric hindrance, but the overall distribution confirms that the generated structures are geometrically reasonable.

### Comparison with experimental structures

**Fig. 4:**
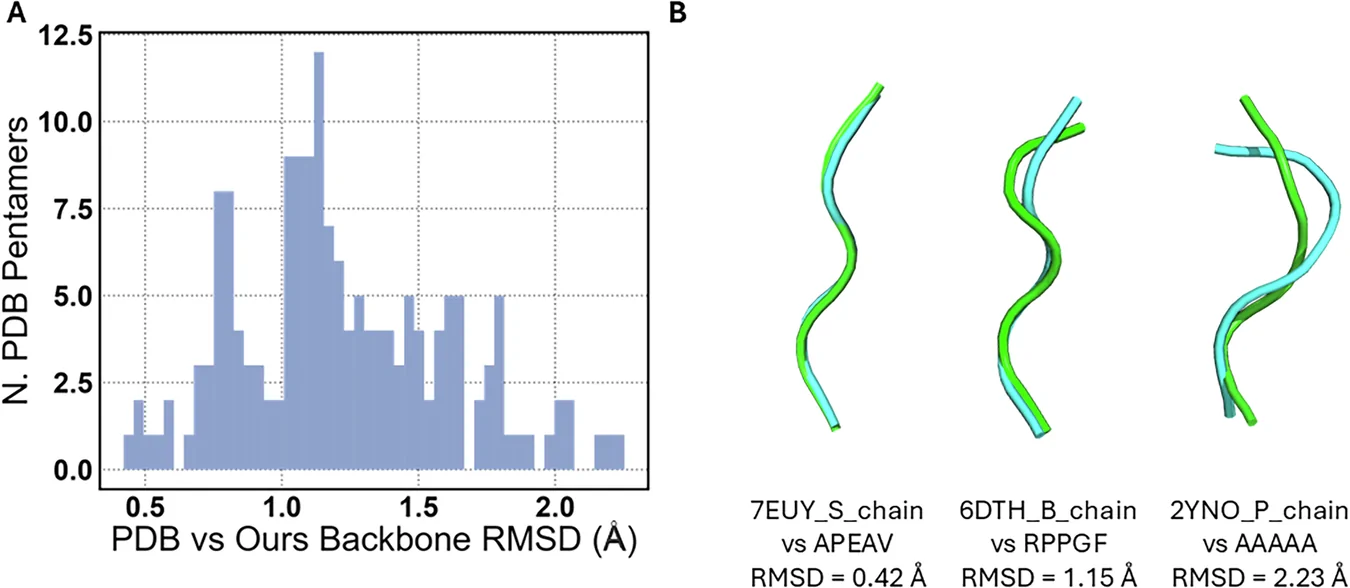
Comparison of predicted peptide conformers to experimentally determined PDB structures. (**A**) RMSD values between the best conformer (out of 10) generated for each pentapeptide and its corresponding experimental structure from the PDB. (**B**) Best, median, and worst cases: Left, near-perfect alignment between the modeled APEAV and the corresponding structure from PDB entry 7EUY S chain (RMSD = 0.43 Å); Center, median alignment RMSD between RPPGF and PDB entry 6DTH chain B; Right, a more flexible sequence (AAAAA) compared to 2YNO chain P, showing a larger deviation (RMSD = 2.22 Å).

We benchmarked the library against experimentally observed pentapeptide fragments extracted from the Protein Data Bank (PDB) using symmetry-aware backbone heavy-atom RMSD. The comparison gave us a mean best-conformer RMSD of 1.19 Å and a median of 1.12 Å, with 78.0% of cases falling below 1.5 Å and 96.1% falling below 2.0 Å (Fig. 4A,B). This analysis indicates that the library captures experimentally relevant local peptide conformations at the backbone level. We interpret these RMSD results as evidence that the generated conformers fall within a realistic structural range for experimentally observed pentapeptide backbones. This benchmark should not be interpreted as a test of whether the library predicts a single environment-specific conformation, particularly side-chain orientations that may be influenced by crystal packing, receptor binding, solvent exposure, pH, or other experimental constraints. Instead, the comparison indicates that the conformer ensembles provide reasonable structural coverage of local peptide conformations observed in the PDB. We did not benchmark against experimental observables such as NOEs or chemical shifts, as such comparisons require system-specific ensembles and forward models that are beyond the scope of a general-purpose conformer dataset. Accordingly, the present resource is intended as a standardized conformer library rather than an experimentally refined ensemble for any single peptide.

We release an open, automated workflow based on UCSF ChimeraX, Reduce, and RDKit for rapid generation of conformational ensembles of short peptides. The pipeline is reproducible, scales to large libraries, and can be applied beyond pentapeptides to support screening, docking, and machine learning development.

## Usage Notes

The library is intended for high-throughput peptide modeling, conformer benchmarking, virtual screening, and machine-learning workflows. Structures are provided in PDB format and can be used directly with common docking packages such as AutoDock Vina and Glide, as well as visualization tools including PyMOL and UCSF ChimeraX. Users interested in specific peptides or subsets can retrieve only the relevant archive members using the provided index rather than unpacking the full dataset. For studies involving only a small number of peptides, users may find it more practical to generate structures directly with the provided pipeline, whereas the full dataset is intended primarily for high-throughput and standardized large-scale workflows.

For docking and scoring, we recommend evaluating multiple conformers per sequence to account for peptide flexibility. Because many docking engines perform their own internal ligand sampling, the library is especially useful when reproducible external peptide ensembles are desired, for example in benchmarking, prescreening, rescoring, or workflows with limited conformational sampling.

## Data Availability

The dataset supporting this manuscript is publicly available in Zenodo (https://doi.org/10.5281/zenodo.18665396).

## Code availability

All scripts and methods used to generate this dataset, including the automated ChimeraX, Reduce, and RDKit workflow, are available in the GitHub repository: https://github.com/jrcodina/Build_Peptides.git. The repository includes documentation on dependencies and usage instructions to reproduce the pipeline or apply it to different peptide sets.
